# Chromosome constitution and genetic relationships of *Morus* spp. revealed by genomic in situ hybridization

**DOI:** 10.1186/s12870-023-04448-9

**Published:** 2023-09-15

**Authors:** Yahui Xuan, Sheng Wang, Siwei Li, Jianglian Yuan, Qiming Zhou, Ningjia He

**Affiliations:** State Key Laboratory of Resource Insects, Southwest University, Beibei, Chongqing, 400715 China

**Keywords:** *Morus*, Genomic in situ hybridization, Chromosome constitution, Genetic relationships, Mulberry section

## Abstract

**Background:**

Mulberry (*Morus* spp.) is an economically important woody plant, which has been used for sericulture (silk farming) for thousands of years. The genetic background of mulberry is complex due to polyploidy and frequent hybridization events.

**Results:**

Comparative genomic in situ hybridization (cGISH) and self-GISH were performed to illustrate the chromosome constitution and genetic relationships of 40 mulberry accessions belonging to 12 species and three varietas in the *Morus* genus and containing eight different ploidy levels. We identified six homozygous cGISH signal patterns and one heterozygous cGISH signal pattern using four genomic DNA probes. Using cGISH and self-GISH data, we defined five mulberry sections (*Notabilis*, *Nigra*, *Wittiorum*, and *Cathayana*, all contained only one species; and *Alba*, which contained seven closely related species and three varietas, was further divided into two subsections) and proposed the genetic relationships among them. Differential cGISH signal patterns detected in section *Alba* allowed us to refine the genetic relationships among the closely related members of this section.

**Conclusions:**

We propose that GISH is an efficient tool to investigate the chromosome constitution and genetic relationships in mulberry. The results obtained here can be used to guide outbreeding of heterozygous perennial crops like mulberry.

**Supplementary Information:**

The online version contains supplementary material available at 10.1186/s12870-023-04448-9.

## Background

Mulberry (*Morus* spp.) comprises of deciduous trees and shrubs in the family Moraceae. As the main food source of domesticated silkworms (*Bombyx mori*), mulberry has been cultivated in China for over 5,000 years and greatly influenced human civilization along the Silk Road [1]. Further adding to the economic importance of mulberry is the medicinal value of its fruit, leaves, and roots [2–4]. Mulberry originated in the Himalayan foothills and spread to all regions of the world except for Antarctica [5, 6]. The wide environmental adaptability of mulberry also gives it important roles in ecological protection [7, 8].

Natural and artificial hybridization between different mulberry species has produced numerous interspecific hybrids, greatly complicating the genetic background of mulberry, hindering the breeding and efficient utilization of this important crop [9]. Morphological classification of the genus *Morus* was firstly established by Linnaeus in 1753 [10]. Later, Bureau developed a comprehensive taxonomy of *Morus*, using the morphological characteristics of leaves and pistillate catkins to classify *Morus* into five species, 19 varietas, and 13 sub-varietas [11]. Since then, the classification of *Morus* has been revised many times [12–14]. To date, as many as 150 *Morus* species have been reported [9]. Interspecific hybridization and environmental factors such as geographic position interfered with the identification of mulberry phenotypes [9, 15]. Therefore, most of the reported mulberry species should be treated as synonyms or varietas [9, 16]. Even though 10–16 mulberry species are widely recognized and cited [16, 17], classification based on morphology remains controversial.

Molecular markers provide a faster and more reliable system for germplasm characterization and phylogenetic analyses, and are unaffected by environment factors [18, 19]. Muhonja investigated the genetic relationships among 54 mulberry accessions from eight species using genome-wide single nucleotide polymorphism markers and proposed three monophyletic species [15]. Another study used inter-simple sequence repeat and random amplified polymorphic DNA markers to explore the population structure of 19 mulberry genotypes from five species, and concluded that *M. laevigata* was a separate species and *M. latifolia*, *M. bombycis*, *M. alba*, and *M. indica* should be considered as a single species [20]. Directed amplification of minisatellite DNA, amplified fragment-length polymorphism, and simple sequence repeats were also used in phylogenetic analyses of mulberry [21–23]. Nepal and Ferguson recognized 13 *Morus* species based on phylogenetic analyses using internal transcribed spacer (ITS) sequences and chloroplast *trn*L*-trn*F intergenic spacer region [5]. Zeng redefined the classification of *Morus* species based on comprehensive analyses of ITS sequences from 43 mulberry accessions, and proposed eight species (*M. alba*, *M. nigra*, *M. notabilis*, *M. serrata*, *M. celtidifolia*, *M. insignis*, *M. rubra*, and *M. mesozygia*) [16]. In addition, most of the mulberry accessions (belonging to 12 species) collected in China with many morphological polymorphisms were clustered into a single clade B [16]. Based on phylogenetic analyses using ITS sequences, ITS pseudogenes, and chloroplast DNA (cpDNA) sequences, Xuan further separated clade B into three clades and proposed that hybridization between different species played important roles in mulberry evolution [17]. Furthermore, of two recent population genomics analyses focusing on cultivated mulberry, one classified 134 mulberry accessions into three geographical groups and the other classified 155 mulberry accessions into six genetic groups [24, 25]. These studies mainly clarified the population structure of cultivated mulberry species, however, wild mulberry species with abundant morphological diversities still lack reliable classification.

Polyploidy and hybridization play important roles in plant speciation and evolution. Heterosis and polyploidy have also been used to greatly increase the yield of crops and trees [26, 27]. Ten different chromosome numbers with two basic chromosome numbers (× = 7 and ×  = 14) have been identified in different mulberry species [28, 29]. A concerted effort is needed to characterize and evaluate the chromosome constitution and genetic relationships of mulberry. Genomic in situ hybridization (GISH) allows researchers to visualize the genomic organization and evolutionary relationships of polyploid taxa and hybrids [30, 31], and has been used to classify new genera and species [32]. Piperidis used GISH to identify the interspecific hybrids of two closely related species and the intergeneric hybrids of two closely related genera [33]. GISH can also be used to identify genome donors, even those of extinct ancestors [34–36]. Therefore, GISH can provide reliable genome information for breeding programs [37].

In this study, we conducted comparative GISH (cGISH) analyses of 40 mulberry accessions from 12 species and three varietas to investigate their chromosome constitution and genetic relationships. Next, we performed self-GISH and fluorescence in situ hybridization (FISH) using the 25S rDNA sequence as a probe in the homozygous mulberry accessions to construct the self-GISH signal pattern of each species. Our results provide a foundation for future cytogenetic studies and genome information for mulberry breeding programs.

## Results

### Self-GISH in four mulberry species

**Fig. 1:**
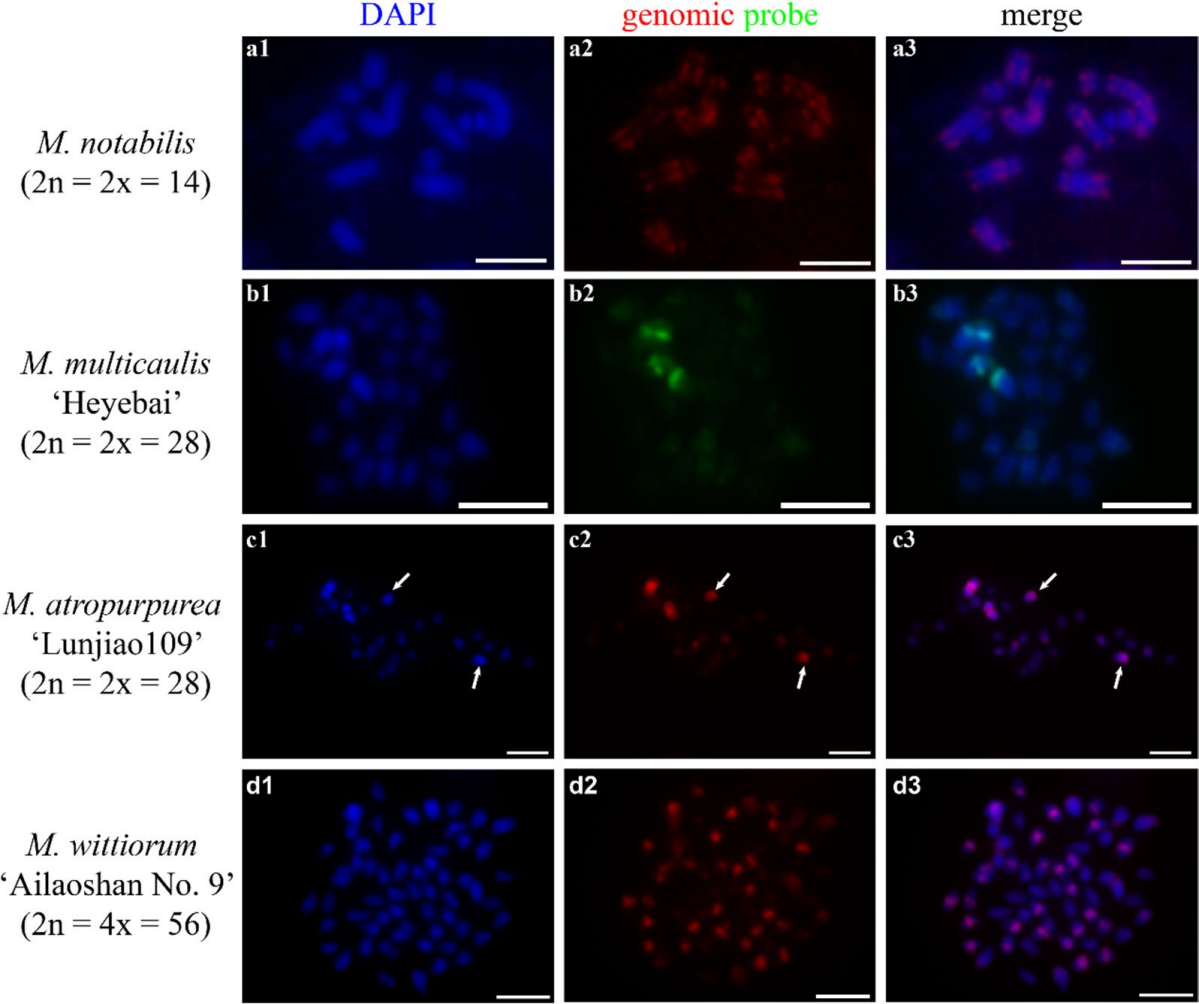
Self-genomic in situ hybridization (self-GISH) signal patterns. *M. notabilis* (**a1-3**), *M. multicaulis* ‘Heyebai’ (**b1-3**), *M. atropurpurea* ‘Lunjiao109’ (**c1-3**), and *M. wittiorum* ‘Ailaoshan No. 9’ (**d1-3**). **a1, b1, c1, d1**: chromosomes counterstained with DAPI. **a2, b2, c2, d2**: self-GISH signals in *M. notabilis* (red), *M. multicaulis* ‘Heyebai’ (green), *M. atropurpurea* ‘Lunjiao109’ (red), and *M. wittiorum* ‘Ailaoshan No. 9’ (red). **a3, b3, c3, d3**: merged signals. Arrows in **c1, c2**, and **c3** indicate chromosome 2 and the signal bands located at the short arms of chromosome 2. Scale bars represent 5 μm

First, we performed self-GISH in four mulberry accessions (*M. notabilis* (2n = 2x = 14), *M. multicaulis* ‘Heyebai’ (2n = 2x = 28), *M. atropurpurea* ‘Lunjiao109’ (2n = 2x = 28), and *M. wittiorum* ‘Ailaoshan No. 9’ (2n = 4x = 56) to investigate the chromosome constitution of these four genomes and their potential to be used as GISH probes. All chromosomes of *M. notabilis* showed clear and intense self-GISH signal bands, except for the smallest chromosome, #7, which showed weak signals (Fig. 1 a1-3). We constructed a karyotype of *M. notabilis* based on the number and position of the signal bands as shown in Fig. 1 a3 (Fig. S1). The self-GISH pattern of *M. multicaulis* ‘Heyebai’ showed that both arms of the deeply DAPI-stained chromosome 1 had bright signal bands at the terminal regions, while the other parts of the chromosome arms had similar-intensity painting signals (Fig. 1 b1-3). The remaining chromosomes showed varying painting signal intensities and telomere signals (Fig. 1 b1-3). The self-GISH pattern of *M. atropurpurea* ‘Lunjiao109’ showed six bright signal bands; two were located at the short arms of chromosome 2, which showed clear primary constrictions (indicated by white arrows), and the other four were located at the terminal regions of the four arms of chromosome 1 (Fig. 1 c1-3). The remaining chromosomes showed relatively weak signals (Fig. 1 c1-3). In *M. wittiorum* ‘Ailaoshan No. 9’, thirty-two chromosomes showed intense signal bands and 24 chromosomes showed weak signal bands (Fig. 1 d1-3). These results suggested that these four mulberry accessions were homozygous and their genomic DNA could be used as GISH probes.

### Comparative GISH in different mulberry species

**Fig. 2:**
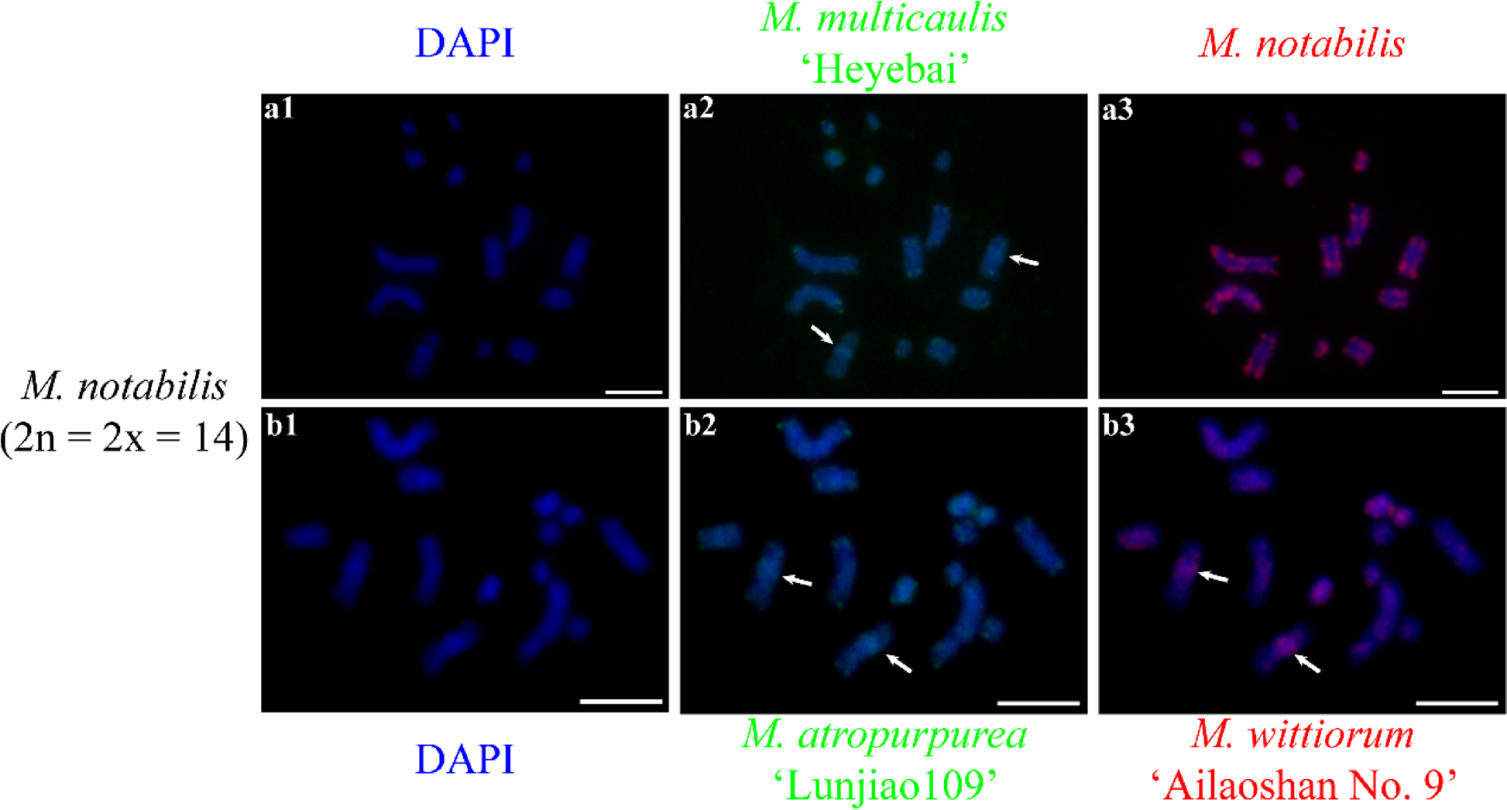
Comparative genomic in situ hybridization (cGISH) signal pattern 1 detected in *M. notabilis.*** a1, a2, a3**: cGISH signal patterns in *M. notabilis* using genomic DNA of *M. multicaulis* ‘Heyebai’ and *M. notabilis* as probes. **b1, b2, b3**: cGISH signal patterns in *M. notabilis* using genomic DNA of *M. atropurpurea* ‘Lunjiao109’ and *M. wittiorum* ‘Ailaoshan No. 9’ as probes. Weak signals detected using genomic DNA of *M. multicaulis* ‘Heyebai’ (**a2**), *M. atropurpurea* ‘Lunjiao109’ (**b2**), and *M. wittiorum* ‘Ailaoshan No. 9’ (**b3**) were adjusted using Adobe Photoshop CS6 to facilitate observation. Arrows in **a2, b2,** and **b3** indicate the intense signal bands collocated with the heterochromatin region in one pair of middle-length chromosomes. Scale bars represent 5 μm

**Fig. 3:**
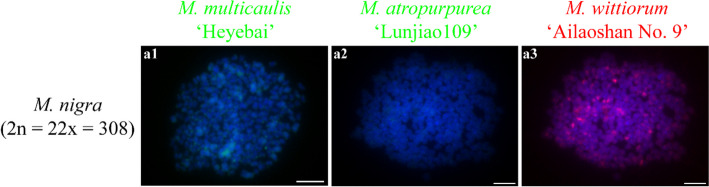
Comparative genomic in situ hybridization (cGISH) signal pattern 2 detected in *M. nigra*. cGISH signal patterns in *M. nigra* using genomic DNA of *M. multicaulis* ‘Heyebai’ (**a1**), *M. atropurpurea* ‘Lunjiao109’ (**a2**), and *M. wittiorum* ‘Ailaoshan No. 9’ (**a3**) as probes. Scale bars represent 5 μm

**Fig. 4:**
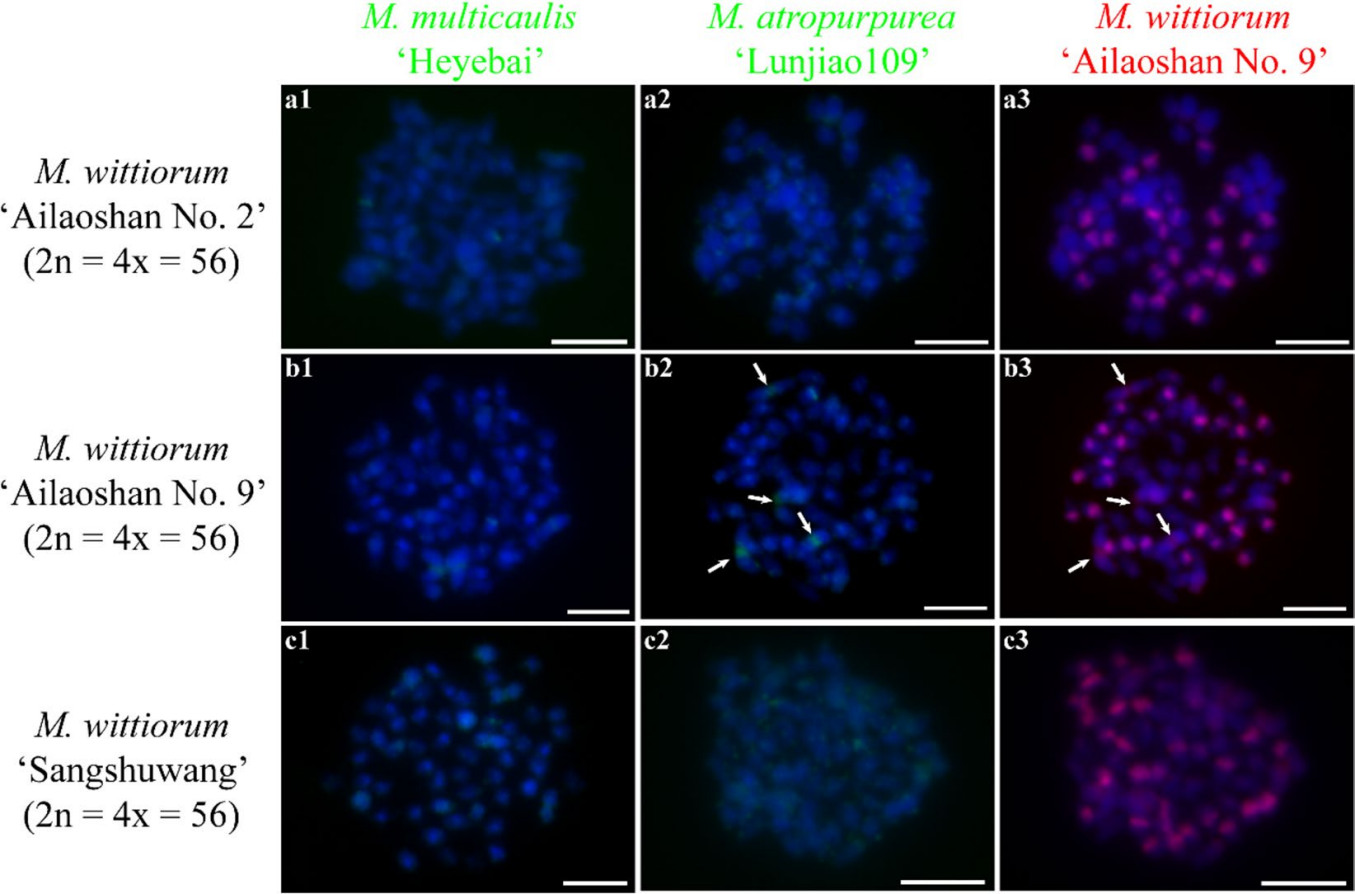
Comparative genomic in situ hybridization (cGISH) signal pattern 3 detected in three *M. wittiorum* accessions. cGISH signal patterns in *M. wittiorum* ‘Ailaoshan No. 2’ (**a1-3**), *M. wittiorum* ‘Ailaoshan No. 9’ (**b1-3**), and *M. wittiorum* ‘Sangshuwang’ (**c1-3**) using genomic DNA of *M. multicaulis* ‘Heyebai’, *M. atropurpurea* ‘Lunjiao109’, and *M. wittiorum* ‘Ailaoshan No. 9’ as probes. Arrows in b2 and b3 indicate the chromosome 1. Scale bars represent 5 μm

**Fig. 5:**
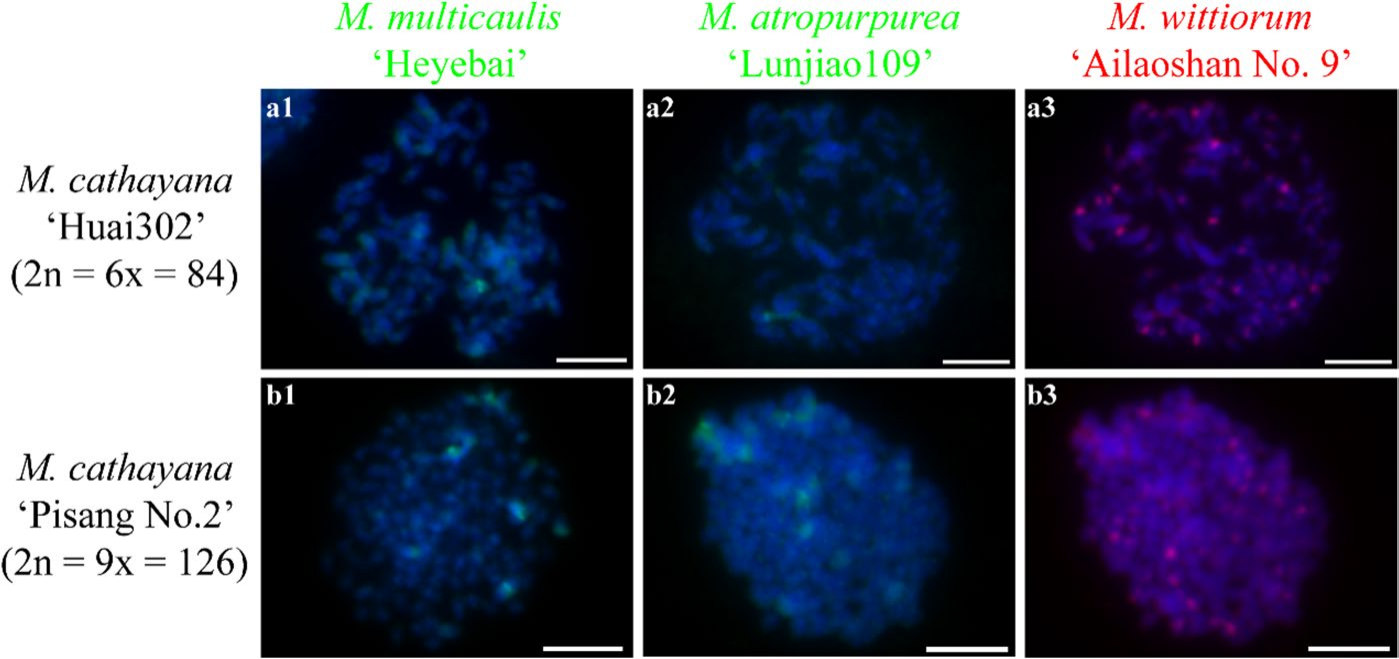
Comparative genomic in situ hybridization (cGISH) signal pattern 4 detected in two *M. cathayana* accessions. cGISH signal patterns in *M. cathayana* ‘Huai302’ (**a1-3**) and *M. cathayana* ‘Pisang No. 2’ (**b1-3**) using genomic DNA of *M. multicaulis* ‘Heyebai’, *M. atropurpurea* ‘Lunjiao109’, and *M. wittiorum* ‘Ailaoshan No. 9’ as probes. Scale bars represent 5 μm

**Fig. 6:**
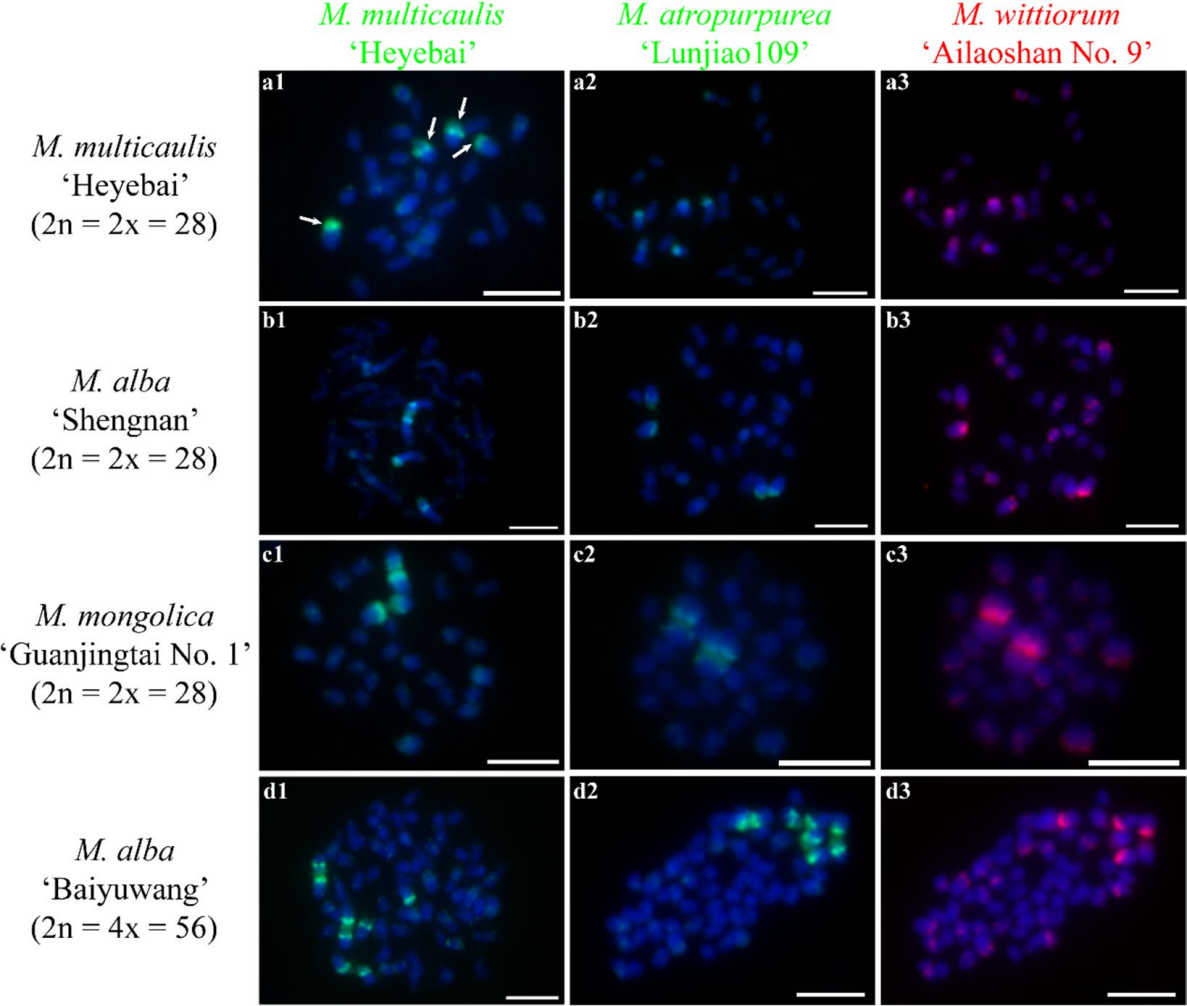
Comparative genomic in situ hybridization (cGISH) signal pattern 5 detected in four mulberry accessions. cGISH signal patterns in *M. multicaulis* ‘Heyebai’ (**a1-3**), *M. alba* ‘Shengnan’ (**b1-3**), *M. mongolica* ‘Guanjingtai No. 1’ (**c1-3**), and *M. alba* ‘Baiyuwang’ (**d1-3**) using genomic DNA of *M. multicaulis* ‘Heyebai’, *M. atropurpurea* ‘Lunjiao109’, and *M. wittiorum* ‘Ailaoshan No. 9’ as probes. Arrows in **a1** indicate four arms of chromosome 1. Scale bars represent 5 μm

**Fig. 7:**
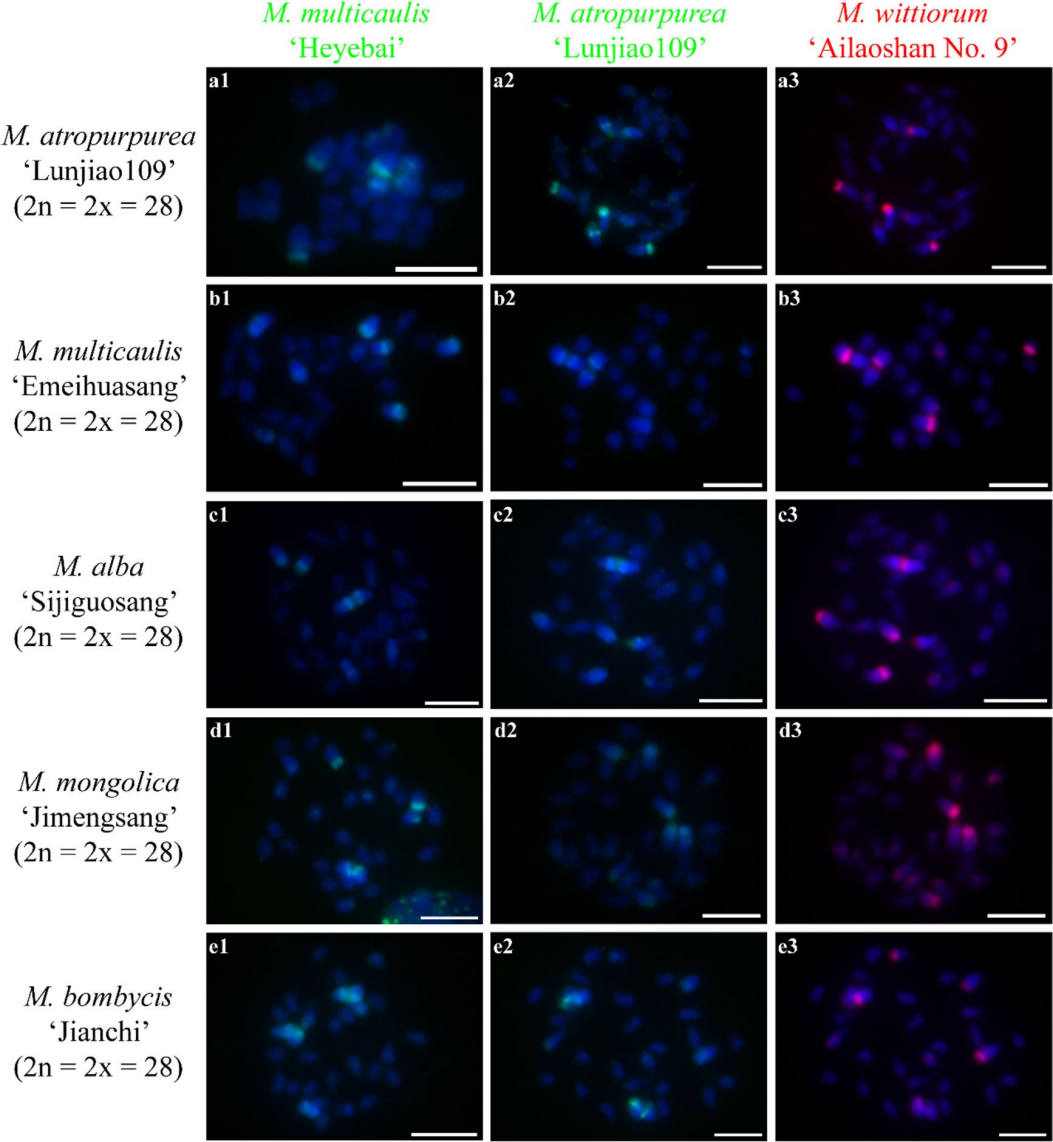
Comparative genomic in situ hybridization (cGISH) signal pattern 6 detected in five mulberry accessions. cGISH signal patterns in *M. atropurpurea* ‘Lunjiao109’ (**a1-3**), *M. multicaulis* ‘Emeihuasang’ (**b1-3**), *M. alba* ‘Sijiguosang’ (**c1-3**), *M. mongolica* ‘Jimengsang’ (**d1-3**), and *M. bombycis* ‘Jianchi’ (**e1-3**) using genomic DNA of *M. multicaulis* ‘Heyebai’, *M. atropurpurea* ‘Lunjiao109’, and *M. wittiorum* ‘Ailaoshan No. 9’ as probes. Scale bars represent 5 μm

**Fig. 8:**
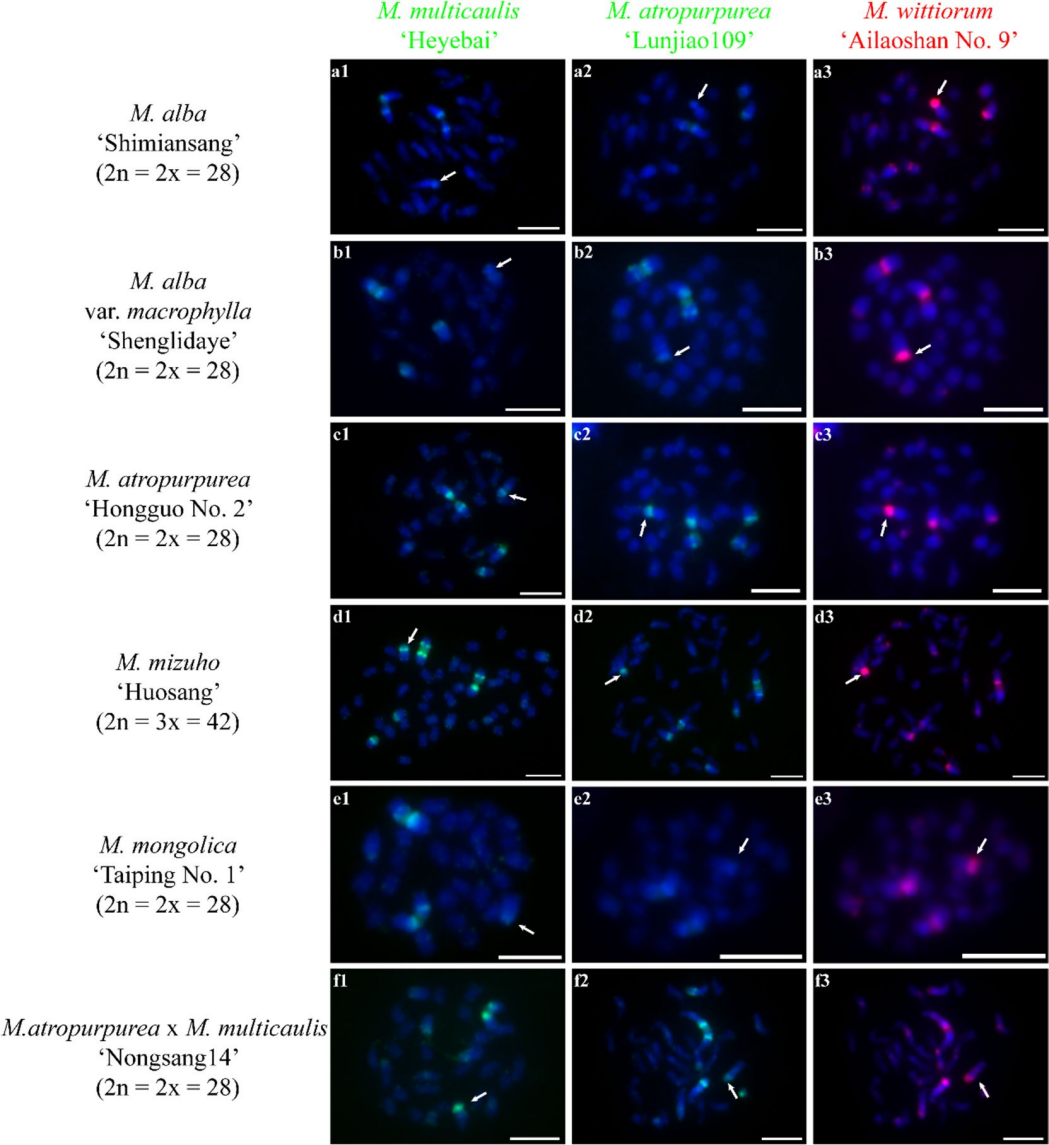
Comparative genomic in situ hybridization (cGISH) signal pattern 7 detected in six mulberry accessions. cGISH signal patterns in *M. alba* ‘Shimiansang’ **(a1-3)**, *M. alba* var. *macrophylla* ‘Shenglidaye’ (**b1-3**), *M. atropurpurea* ‘Hongguo No. 2’ (**c1-3**), *M. mizuho* ‘'Huosang’ (**d1-3**), *M. mongolica* ‘Taiping No. 1’ (**e1-3**), and *M. atropurpurea* x *M. multicaulis* ‘Nongsang14’ (**f1-3**) using genomic DNA of *M. multicaulis* ‘Heyebai’, *M. atropurpurea* ‘Lunjiao109’, and *M. wittiorum* ‘Ailaoshan No. 9’ as probes. Arrows indicate unpaired chromosome 2 with intense signal bands at the short arms. Scale bars represent 5 μm

Next, we investigated the chromosome constitutions of mulberry species through cGISH using the genomic DNA of *M. notabilis*, *M. multicaulis* ‘Heyebai’, *M. atropurpurea* ‘Lunjiao109’, and *M. wittiorum* ‘Ailaoshan No. 9’ as probes without blocking DNA. We detected seven distinct cGISH signal patterns and one ungrouped signal mix among 40 mulberry accessions. We detected cGISH signal pattern 1 in *M. notabilis* (Fig. 2). The signal patterns of the *M. multicaulis* ‘Heyebai’ and *M. atropurpurea* ‘Lunjiao109’ genomic DNA probes were similar in *M. notabilis*: all chromosomes had weak signals and we clearly observed telomere signals at both ends of all chromosomes (Fig. 2 a2, b2). In addition, one pair of relatively bright signal bands collocated with the deeply DAPI-stained heterochromatin regions on one pair of middle-length chromosomes (indicated by the white arrows). The *M. notabilis* genomic DNA probe showed the same signal pattern as described above in Fig. 1 a3 (Fig. 2 a3). The *M. wittiorum* ‘Ailaoshan No. 9’ genomic DNA probe showed relatively bright signals in one pair of middle length chromosomes (indicated by white arrows in Fig. 2 b3) and in chromosomes 5 and 6, while the remaining chromosomes showed weak signals (Fig. 2 b3). The cGISH signal intensity using the *M. notabilis* genomic DNA probe was very weak in all other mulberry accessions (data not shown). We detected cGISH signal pattern 2 in *M. nigra* (Fig. 3). In brief, *M. multicaulis* ‘Heyebai’ and *M. atropurpurea* ‘Lunjiao109’ genomic DNA probes had weak signals in all *M. nigra* chromosomes and deeply DAPI-stained chromosomes collocated with relatively bright signals (Fig. 3 a1, a2). The *M. wittiorum* ‘Ailaoshan No. 9’ genomic DNA probe showed intense signals across all *M. nigra* chromosomes and stronger signal bands at some chromosomes (Fig. 3 a3). We detected cGISH signal pattern 3 in *M. wittiorum* ‘Ailaoshan No. 2’, *M. wittiorum* ‘Ailaoshan No. 9’, and *M. wittiorum* ‘Sangshuwang’ (Fig. 4). *M. multicaulis* ‘Heyebai’ and *M. atropurpurea* ‘Lunjiao109’ genomic DNA probes showed strong signals mainly at chromosomes 1 and 2, and weak signals at all other chromosomes in these three mulberry accessions (Fig. 4 a1, a2, b1, b2, c1, and c2). We detected telomere signals in all chromosomes using genomic DNA of *M. atropurpurea* ‘Lunjiao109’ as the probe (Fig. 4 a2, b2, and c2). The *M. wittiorum* ‘Ailaoshan No. 9’ genomic DNA probe showed signals at 28 to 32 chromosomes among these tetraploid *M. wittiorum* accessions, with bright signal bands in the putative centromeric regions (Fig. 4 a3, b3, and c3). The remaining chromosomes showed medium intensity signal bands at the putative centromeric regions (Fig. 4 a3, b3, and c3), including chromosome 1, which collocated with the bright cGISH signals produced using *M. multicaulis* ‘Heyebai’ and *M. atropurpurea* ‘Lunjiao109’ genomic DNA as probes (indicated by arrows in Fig. 4 b2 and b3). We detected cGISH signal pattern 4 in *M. cathayana* ‘Huai302’ and *M. cathayana* ‘Pisang No. 2’ (Fig. 5). *M. multicaulis* ‘Heyebai’ and *M. atropurpurea* ‘Lunjiao109’ genomic DNA probes showed similar signal patterns; signal intensities varied among the chromosomes and some chromosomes had bright signal bands (Fig. 5 a1, a2, b1, and b2). All chromosomes showed high- or medium-intensity signals from the *M. wittiorum* ‘Ailaoshan No. 9’ genomic DNA probe at the centromere regions (Fig. 5 a3 and b3). We detected cGISH signal pattern 5 in *M. multicaulis* ‘Heyebai’, *M. alba* ‘Shengnan’, *M. mongolica* ‘Guanjingtai No. 1’, and *M. alba* ‘Baiyuwang’ (Fig. 6). In the diploid mulberry accessions (*M. multicaulis* ‘Heyebai’, *M. alba* ‘Shengnan’, and *M. mongolica* ‘Guanjingtai No. 1’), the four deeply DAPI-stained arms of a single chromosome were hybridized with bright signal bands at the terminal ends using *M. multicaulis* ‘Heyebai’ and *M. atropurpurea* ‘Lunjiao109’ genomic DNA probes (Fig. 6 a1, a2, b1, b2, c1, and c2); this was identified as chromosome 1 based on the chromosomal morphology and further verified below (indicated by arrows in Fig. 6 a1). The remaining chromosomes showed weak signals throughout, and we detected weak telomere signals in all the mulberry accessions using *M. multicaulis* ‘Heyebai’ and *M. atropurpurea* ‘Lunjiao109’ genomic DNA probes (Fig. 6 a1, a2, b1, b2, c1, and c2). The *M. wittiorum* ‘Ailaoshan No. 9’ genomic DNA probe produced bright signal bands at one arm of chromosome 1 and medium intensity painting signals at another arm of chromosome 1 (Fig. 6 a3, b3, and c3). Eight to ten chromosomes had medium intensity signals and the remaining chromosomes had weak signals with the *M. wittiorum* ‘Ailaoshan No. 9’ probe (Fig. 6 a3, b3, and c3). In the tetraploid mulberry accession *M. alba* ‘Baiyuwang’, the signals were two-fold higher than the signals detected in *M. multicaulis* ‘Heyebai’, *M. alba* ‘Shengnan’, and *M. mongolica* ‘Guanjingtai No. 1’ (Fig. 6 d1-d3). We detected cGISH signal pattern 6 in *M. atropurpurea* ‘Lunjiao109’, *M. atropurpurea* ‘Wuhedashi’, *M. multicaulis* ‘Emeihuasang’, *M. multicaulis* ‘Zhuangelou’, *M. alba* ‘Sijiguosang’, *M. alba* ‘Kanwa’, *M. alba* ‘Zhenzhubai’, *M. alba* var. *pendula* ‘Chuisang’, *M. alba* var. *macrophylla* ‘Dayezaoshengsang’, *M. mongolica* ‘Jimengsang’, *M. mongolica* var. *diabolica* ‘Taiping No. 5’, *M. mongolica* var. *diabolica* ‘Taiping No. 6’, *M. bombycis* ‘Jianchi’, and *M. bombycis* ‘Xinjianchi’ (Fig. 7 and S2). *M. multicaulis* ‘Heyebai’ and *M. atropurpurea* ‘Lunjiao109’ genomic DNA probes produced similar signal patterns in all mulberry accessions, with bright signals at chromosomes 1 and 2 (Fig. 7 and S2). The remaining chromosomes had weak signals and showed telomere signals (Fig. 7 and S2). We observed bright signals at chromosomes 1 and 2 using the *M. wittiorum* ‘Ailaoshan No. 9’ genomic DNA probe, and varying signal intensity at the other chromosomes (Fig. 7 and S2). We detected cGISH signal pattern 7 in *M. alba* ‘Shimiansang’, *M. alba* var. *macrophylla* ‘Shenglidaye’, *M. atropurpurea* ‘Hongguo No. 2’, *M. mizuho* ‘Huosang’, *M. mongolica* ‘Taiping No. 1’, and *M. atropurpurea* × *M. multicaulis* ‘Nongsang No. 14’ (Fig. 8). The signal patterns of the *M. multicaulis* ‘Heyebai’, *M. atropurpurea* ‘Lunjiao109’, and *M. wittiorum* ‘Ailaoshan No. 9’ genomic DNA probes were similar to patterns 5 and 6, but differed at unpaired chromosome 2, and we detected bright bands at the terminal end of larger chromosome 2 (indicated by white arrows in Fig. 8).

Nine mulberry accessions contained heterozygous cGISH signals, which were distinct from the seven cGISH signal patterns described above (Fig. S3). All three *M. caustralis* accessions had heterozygous signal patterns. We detected the brightest signal bands in unpaired chromosome 1 (indicated by red arrows in Fig. S3 a1-3) and unpaired chromosome 2 with intense signals at the terminal regions (indicated by white arrows in Fig. S3 a1-3) in *M. caustralis* ‘Jisang’ using *M. multicaulis* ‘Heyebai’, *M. atropurpurea* ‘Lunjiao109’, and *M. wittiorum* ‘Ailaoshan No. 9’ genomic DNA as probes. In *M. caustralis* ‘Taiping No. 3’, *M. multicaulis* ‘Heyebai’ and *M. atropurpurea* ‘Lunjiao109’ genomic DNA produced intense signal bands at two arms of chromosome 1 (indicated by red arrows in Fig. S3 b1, b2), and five medium-intensity signal bands (Fig. S3 b1, b2). We detected bright signal bands at 22 chromosomes using *M. wittiorum* ‘Ailaoshan No. 9’ genomic DNA as probe, and the remaining chromosomes showed weak signals (Fig. S3 b3). In *M. caustralis* ‘Longling No. 2’, we detected two bright signal bands using *M. multicaulis* ‘Heyebai’ and *M. atropurpurea* ‘Lunjiao109’ genomic DNA as probes (Fig. S3 c1, c2). We detected bright signal bands at 34 chromosomes using *M. wittiorum* ‘Ailaoshan No. 9’ genomic DNA as probe, and the other chromosomes showed weak signals (Fig. S3 c3). In *M. alba* ‘Agentingsang’, we detected unpaired signals at chromosome 1 using *M. multicaulis* ‘Heyebai’, *M. atropurpurea* ‘Lunjiao109’, and *M. wittiorum* ‘Ailaoshan No. 9’ genomic DNA as probes. One chromosome 1 showed one intense signal bands using these three probes (indicated by solid red arrows), and the other chromosome 1 showed two intense signal bands using *M. multicaulis* ‘Heyebai’ and *M. atropurpurea* ‘Lunjiao109’ genomic DNA as probes and medium-intensity signals using *M. wittiorum* ‘Ailaoshan No. 9’ genomic DNA as probe (indicated by dashed red arrows). The pair of chromosomes 2, with clear primary constrictions, showed paired signal bands (indicated by white arrows in Fig. S3 d2, d3). In *M. laevigata* ‘Taiwanchaochangguo’, we detected signals with varying intensity and telomere signals using *M. multicaulis* ‘Heyebai’ and *M. atropurpurea* ‘Lunjiao109’ genomic DNA as probes (Fig. S3 e1, e2). The *M. wittiorum* ‘Ailaoshan No. 9’ genomic DNA probe produced 18 intense centromere signal bands and several weak signal bands in this accession (Fig. S3 e3). In *M. wittiorum* ‘Yun6muben’, six chromosomes had bright signals and the other chromosomes had weak signals using *M. multicaulis* ‘Heyebai’ and *M. atropurpurea* ‘Lunjiao109’ genomic DNA as probes, and we detected telomere signals in all chromosomes (Fig. S3 f1, f2). We observed five intense signal bands and 18 medium-intensity signal bands using *M. wittiorum* ‘Ailaoshan No. 9’ genomic DNA as probe (Fig. S3 f3). In *M. wittiorum* ‘Shuisang’, we detected signal bands of varying intensity using *M. multicaulis* ‘Heyebai’ and *M. atropurpurea* ‘Lunjiao109’ genomic DNA as probes, and one unpaired chromosome 1 had bright signal bands (Fig. S3 g1, g2). Using *M. wittiorum* ‘Ailaoshan No. 9’ genomic DNA as probe, we detected 15 intense centromere signal bands and several weak signal bands (Fig. S3 g3). In *M. wittiorum* ‘Sangshuwang No. 8’, the *M. multicaulis* ‘Heyebai’ and *M. atropurpurea* ‘Lunjiao109’ genomic DNA probes produced intense signal bands at both arms of one chromosome 1, and signals of varying intensity at all other chromosomes (Fig. S3 h1, h2), and the *M. wittiorum* ‘Ailaoshan No. 9’ genomic DNA probe produced 20 intense signal bands (Fig. S3 h3). In *M. cathayana* ‘Huasang’, two arms of one chromosome 1 had bright signal bands using *M. multicaulis* ‘Heyebai’ and *M. atropurpurea* ‘Lunjiao109’ genomic DNA as probes (Fig. S3 i1, i2). We observed signal bands of varying intensity with the *M. wittiorum* ‘Ailaoshan No. 9’ genomic DNA probe (Fig. S3 i3).

### Species-specific GISH signal patterns of each mulberry species

**Fig. 9:**
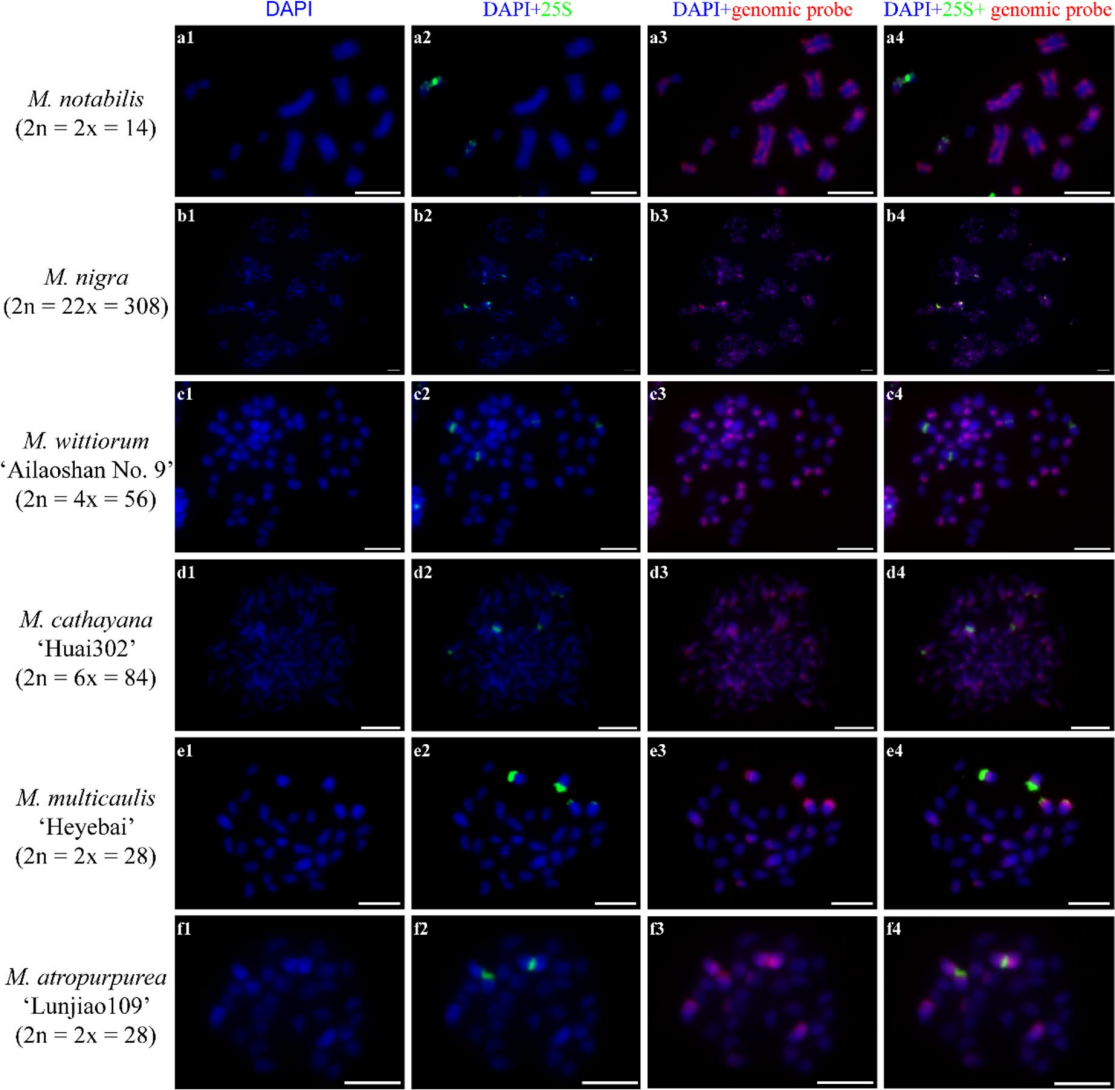
Self-genomic in situ hybridization (self-GISH) and fluorescence in situ hybridization (FISH) signal patterns detected in six mulberry accessions. **1a-f**: chromosomes counterstained with DAPI. **2a-f**: FISH signal patterns using the 25S rDNA sequence as probe. **3a-f**: self-GISH signal patterns. **4a-f**: merged self-GISH and FISH signal patterns. Scale bars represent 5 μm

Based on the cGISH patterns described above, we selected 12 homozygous mulberry accessions from nine *Morus* species and three varietas for self-GISH to explore the species-specific GISH signal patterns. We performed FISH experiments using 25S rDNA as probe to identify chromosome 5 and chromosome 7 in *M. notabilis* and chromosome 1 in the other mulberry accessions (Fig. 9 and S4) [28]. In *M. notabilis*, the self-GISH signal pattern was the same as that shown in Fig. 1 a1-3, and chromosome 5 and chromosome 7 had bright and weak 25S rDNA signal bands at the terminal ends, respectively (Fig. 9 a1-4). In *M. nigra*, the self-GISH signal pattern showed clear and intense telomere signals at all chromosomes and putative centromere signals at most of the chromosomes, and the 25S rDNA probe signals collocated with intense self-GISH signal bands at the deeply DAPI-stained chromosomes (Fig. 9 b1-4). The self-GISH signal pattern of *M. wittiorum* ‘Ailaoshan No. 9’ was the same as that shown in Fig. 1 d1-3, and four signal bands of the 25S rDNA probe collocated with four medium-intensity self-GISH signal bands at the middle regions of chromosome 1 (Fig. 9 c1-4). In *M. cathayana* ‘Huai302’, the self-GISH signal pattern differed from the patterns using the *M. multicaulis* ‘Heyebai’, *M. atropurpurea* ‘Lunjiao109’, and *M. wittiorum* ‘Ailaoshan No. 9’ genomic DNA probes (Fig. 9 d1-4). We detected putative centromere signal bands at all chromosomes and strong painting signals. Terminal or central 25S rDNA bands collocated with the strong painting signals at chromosome 1. In *M. multicaulis* ‘Heyebai’, the self-GISH signal pattern was the same as that shown in Fig. 1, and the 25S rDNA signals (two bright and two weak) collocated with the four bright signal bands at the four deeply DAPI-stained arms of chromosome 1 (Fig. 9 e1-4). In *M. alba* ‘Baiyuwang’, the self-GISH signal pattern was similar to that from the *M. multicaulis* ‘Heyebai’ and *M. atropurpurea* ‘Lunjiao109’ genomic DNA probes; bright genomic DNA probe signals collocated with the 25S rDNA probe signal bands at the pericentromeric regions of chromosome 1, and we detected weak signals at the other chromosomes (Fig. S4 a1-4). In *M. atropurpurea* ‘Lunjiao109’, the self-GISH signal pattern was the same as that shown in Fig. 1 c1-3, and the 25S rDNA probe hybridized in the middle regions of chromosome 1 (Fig. 9 f1-4). In *M. alba* var. *pendula* ‘Chuisang’, *M. alba* var. *macrophylla* ‘Dayezaoshengsang’, *M. bombycis* ‘Jianchi’, *M. mongolica* ‘Jimengsang’, and *M. mongolica* var. *diabolica* ‘Taiping No. 5’, the self-GISH and 25S rDNA signal patterns were similar to those detected in *M. atropurpurea* ‘Lunjiao109’. Chromosomes 1 and 2 showed bright signal bands, while the signal intensity varied in the other chromosomes. The 25S rDNA probe hybridized at the pericentromeric regions of chromosome 1, which contained the strongest self-GISH signals (Fig. S4 b1-4, c1-4, d1-4, e1-4, f1-4).

## Discussion

Mulberry has high economic and ecological value. As the region of origin, China contains the greatest diversity of mulberry species in the world [5, 9]. Although mulberry species throughout China have been studied morphologically and cytogenetically, the use of more precise techniques, such as GISH, will help us to better understand the chromosome constitution and genetic relationships among mulberry species.

### Chromosome constitution of mulberry elucidated by GISH

GISH is an efficient method to investigate chromosome constitution and evolutionary relationships [30, 31, 38]. Here, we performed self-GISH and cGISH experiments to investigate the chromosome constitution of 40 mulberry accessions belonging to 12 out of the 15 morphologically distinct *Morus* species distributed in China and containing eight ploidy levels. First, the self-GISH results of *M. notabilis*, *M. multicaulis* ‘Heyebai’, *M. atropurpurea* ‘Lunjiao109’, and *M. wittiorum* ‘Ailaoshan No. 9’ showed different type of paired and informative signal patterns (Fig. 1), suggesting a high level of homozygosity at the chromosome level. This suggestion can be supported by genomic data of *M. notabilis* and *M. multicaulis* ‘Heyebai’ [1, 24]. Thus, we used these four mulberry accessions as the genomic DNA probes in our GISH experiments.

Using the cGISH signals from the *M. multicaulis* ‘Heyebai’, *M. atropurpurea* ‘Lunjiao109’, and *M. wittiorum* ‘Ailaoshan No. 9’ genomic DNA probes, we identified six homozygous cGISH signal patterns, suggesting 25 mulberry accessions from nine species and three varietas were homozygous, including polyploid mulberry accessions (triploid *M. atropurpurea* ‘Wuhedashi’; tetraploid *M. alba* ‘Baiyuwang’, *M. wittiorum* ‘Ailaoshan No. 2’, *M. wittiorum* ‘Ailaoshan No. 9’, and *M. wittiorum* ‘Sangshuwang’; hexaploid *M. cathayana* ‘Huai302’; nonuploid *M. cathayana* ‘Pisang No. 2’; and 22-ploid *M. nigra*). Similar results were identified in other species using GISH technique [38]. These results indicate that there are many homozygous mulberry accessions despite the common occurrence of spontaneous and artificial hybridization [17, 39], which is consistent with the similar genome constitutions obtained by genome resequencing [24, 25]. In addition, we identified six mulberry accessions with heterozygous cGISH signal pattern 7 and nine mulberry accessions with ungrouped cGISH signals as interspecific hybrids. Heterozygous signals in triploid *M. mizuho* ‘Huosang’, *M. australis* ‘Taiping No. 3’, *M. wittiorum* ‘Shuisang’, and *M. laevigata* ‘Taiwanchaochangguo’; tetraploid *M. cathayana* ‘Huasang’ and *M. wittiorum* ‘Sangshuwang No. 8’; and pentaploid *M. australis* ‘Longling No. 2’, suggested that they were all allopolyploids. Complex genetic background and evolutionary process of mulberry were suggested here and can be confirmed by the previous reports [17, 35].

Because we obtained highly similar cGISH signal patterns from the *M. multicaulis* ‘Heyebai’ and *M. atropurpurea* ‘Lunjiao109’ genomic DNA probes in all mulberry accessions, we propose that all the cGISH experiments performed here can be done just using two genomic DNA probes (*M. multicaulis* ‘Heyebai’ or *M. atropurpurea* ‘Lunjiao109’ combined with *M. wittiorum* ‘Ailaoshan No. 9’) without blocking DNA. Thus, GISH is a simple and powerful tool to analyze the chromosome constitution of mulberry, even in the 22-ploid *M. nigra*.

### Cytogenetic classification of mulberry

Among the six homozygous cGISH signal patterns identified here, four (patterns 1, 2, 3, and 4) occurred in only one morphologically distinct mulberry species. This phenomenon was also observed in other plants, such as banana (*Musa*) [40], *Allium* [35], and *Kengyilia* [41]. Therefore, we classified these four species into four sections (Table 1). Section *Notabilis* only contained *M. notabilis*; the main characteristics were the self-GISH signal pattern, with signal bands across all chromosomes. Section *Nigra* showed the main characteristics obtained from the self-GISH in *M. nigra*, with dominant telomere signals at all chromosomes and centromere signals at most of the chromosomes. The main characteristics of Section *Wittiorum* were obtained from the self-GISH signal pattern of *M. wittiorum* ‘Ailaoshan No. 9’, which showing about half of the chromosomes having high-intensity putative centromere signal bands and the other half having medium-intensity signal bands. Dominant bright putative centromere signal bands at all chromosomes were detected from the self-GISH in *M. cathayana* ‘Huai302’. This section was named as *Cathayana* after the species name *M. cathayana*. Signal patterns 5 and 6 were similar. We grouped the mulberry accessions with cGISH signal patterns 5 and 6 into section *Alba*, named after the most representative species in this section, *M. alba*. The biggest difference between cGISH signal pattern 5 and 6 was the presence/absence of intense signal bands at the terminal ends of chromosome 2. Based on this difference, we further divided section *Alba* into two subsections (Table 1). Subsection 1 corresponded to cGISH signal pattern 5 and contained three species, *M. alba*, *M. multicaulis*, and *M. mongolica* (Table 1). The main characteristics were from the self-GISH signal patterns using the *M. multicaulis* ‘Heyebai’ genomic DNA probe and the cGISH signal patterns using the *M. multicaulis* ‘Heyebai’ and *M. atropurpurea* ‘Lunjiao109’ genomic DNA probes, with bright signal bands on the both arms of chromosome 1 and medium-intensity signals on the other chromosomes. Subsection 2 corresponded to cGISH signal pattern 6 and contained five species and three varietas, *M. alba*, *M. multicaulis*, *M. atropurpurea*, *M. mongolica*, *M. bombycis*, *M. alba* var. *pendula*, *M. alba* var. *macrophylla*, and *M. mongolica* var. *diabolica* (Table 1). The main characteristics were from the self-GISH signal patterns using the *M. atropurpurea* ‘Lunjiao109’ genomic DNA probe and the cGISH signal patterns using the *M. multicaulis* ‘Heyebai’ and *M. atropurpurea* ‘Lunjiao109’ genomic DNA probes, with bright signal bands on the four arms of chromosomes 1 and 2, and medium-intensity painting signals on the other chromosomes.

**Table 1:** Cytological classification of mulberry based on cGISH and self-GISH signal patterns

Section name	Subsection name	Signal pattern name	Species name	Suggested classification ^a^	Ploidy level ^b^	Major characteristics
*Notabilis*	N/A	Signal pattern 1	*M. notabilis*	*M. yunnanensis*	2x	Signal bands at all the chromosomes
*Nigra*	N/A	Signal pattern 2	*M. nigra*	N/A	22x	Telomere signals at all the chromosomes and centromere signals at most of the chromosomes
*Wittiorum*	N/A	Signal pattern 3	*M. wittiorum*	*M. laevigata*	4x	Half bright centromere signal bands and half medium centromere signal bands
*Cathayana*	N/A	Signal pattern 4	*M. cathayana*	N/A	6x and 9x	Centromere signal bands at all the chromosomes
*Alba*	Subsection 1	Signal pattern 5	*M. alba* *M. multicaulis* *M. mongolica*	*M. mizuho* *M. australis*	2x, 3x, and 4x	Intense signals at chromosome 1
	Subsection 2	Signal pattern 6	*M. alba* *M. multicaulis* *M. atropurpurea* *M. mongolica* *M. bombycis* *M. alba* var. *pendula* *M. alba* var. *macrophylla* *M. mongolica* var. *diabolica*			Intense signals at chromosomes 1 and 2

^a^Suggested classification of mulberry species based on cytological analysis and previous report

^b^Ploidy levels of the homozygous accessions

We detected the heterozygous cGISH signal pattern 7 in four species, one variety, and one hybrid mulberry accession: *M. alba*, *M. atropurpurea*, *M. mongolica*, *M. mizuho*, *M. alba* var. *macrophylla*, and *M. atropurpurea* × *M. multicaulis*. Comparing the main signal difference at chromosome 2 with cGISH signal patterns 5 and 6, the results indicated that the accessions exhibiting cGISH signal pattern 7 were hybrid progenies of the accessions belonging to subsections 1 and 2 in section *Alba*. In addition, many heterozygous accessions showed unique signal patterns. The cGISH signal patterns in *M. australis* ‘Jisang’ and *M. alba* ‘Agentingsang’ were similar to the signals in accessions in section *Alba* and we identified an unpaired chromosome 1 using cGISH. These results suggest there are more subsections in section *Alba*, and that *M. mizuho* and *M. australis* belong in section *Alba* (Table 1). Furthermore, both subsections contain accessions from *M. alba*, *M. multicaulis*, and *M. mongolica* (Table 1), revealing that GISH gives a higher resolution and is more reliable for classifying mulberry accessions than morphological methods. Thus, section *Alba* should contain seven mulberry species and three varietas: *M. alba*, *M. multicaulis*, *M. mongolica*, *M. atropurpurea*, *M. bombycis*, *M. mizuho*, *M. australis*, *M. alba* var. *pendula*, *M. alba* var. *macrophylla*, and *M. mongolica* var. *diabolica* (Table 1).

*M. mesozygia*, *M. insignis*, *M. yunnanensis*, *M. rubra*, *M. serrata*, and *M. celtidifolia*, which were not included in our study, were classified based on phylogenetic analyses using ITS sequences [16]. With the addition of more mulberry accessions from different species, morphological feature analyses, and phylogenetic analyses, more mulberry sections will be classified and the controversy of current mulberry classification will be resolved. In this study, we report a reliable cytogenetic classification system for *Morus*, classifying five mulberry sections and two subsections from 12 species and three varietas.

### Genetic relationships among mulberry species

The five mulberry sections showed variable signal number and intensities, indicating the differentiation and enrichment of species-specific repeat sequences after the divergence of these species [42]. The difference of GISH signal patterns clearly reflected the genetic differences among the five mulberry sections. Little or no cGISH signal was detected at other mulberry accessions using *M. notabilis* genomic probe, suggesting *M. notabilis* is a distinct species and is more distantly related than the other mulberry species used in this study. This result was supported by the cGISH results in other species with long genetic distance [35]. Furthermore, *M. notabilis* and *M. yunnanensis* are the only mulberry species containing the basic chromosome number of 7, whereas all other mulberry species have a basic chromosome number of 14 [17, 25]. Phylogenetic analyses using genomic data and molecular markers supported that *M. notabilis* and *M. yunnanensis* are closely related to each other, but more distantly related to the other mulberry species [16, 17, 25]. We propose that *M. yunnanensis* should be classified into mulberry section *Notabilis* (Table 1).

*M. nigra* (section *Nigra*, 2n = 22x = 308) has the highest chromosome number in the genus *Morus*. We detected intense telomere signals at all chromosomes and centromere signals at most of the chromosomes in *M. nigra*, which differed from the other mulberry accessions. This indicates that *M. nigra* is genetically distinct from the other mulberry accessions, which is supported by its molecular classification based on phylogenetic analyses using ITS sequences, cpDNA sequences, and other molecular markers [16, 17, 43]. *M. wittiorum* (section *Wittiorum*) produces the longest fruit in *Morus*. The section-specific GISH signal pattern consisted of high-intensity centromere signal bands in half of the chromosomes and medium-intensity signal bands in the other half. Our results suggest that *M. wittiorum* is also a distinct species. Phylogenetic analyses of genomic data and ITS sequences validate this hypothesis [17, 25]. *M. laevigata* has morphological characteristics similar to *M. wittiorum*, and they were grouped into the same clade using phylogenetic analyses based on molecular markers [9]. We detected similar heterozygous cGISH signals in triploid *M. laevigata* ‘Taiwanchaochangguo’ and another triploid accession *M. wittiorum* ‘Shuisang’ (Fig. S3 e1-3, g1-3), suggesting that *M. wittiorum* and *M. laevigata* should be classified as a single species in section *Wittiorum* (Table 1) [44]. *M. cathayana* (section *Cathayana*) showed predominant centromere signals at all chromosomes and lacked obvious telomere signals, which differed from the signal patterns in other mulberry species. Thus, *M. cathayana* should be another unique species. The specific accumulation of flavones in leaves of *M. cathayana* accessions also supports that *M. cathayana* should be treated as a unique species [45]. However, *M. cathayana* was grouped with most of the mulberry species distributed in China based on phylogenetic analyses of ITS and cpDNA sequences [16, 17]. This inconsistency between the cytogenetic and molecular classifications of *M. cathayana* indicate that GISH is a more accurate and reliable classification technique. Comparing cGISH signal patterns 2, 3, and 4, we detected predominant centromere signals at most or all of the chromosomes of the species in section *Nigra*, *Wittiorum*, and *Cathayana*, indicating that the three species in these sections are relatively closely related. This was also supported by phylogenetic analyses using molecular markers [46].

The cGISH and self-GISH signal patterns were similar among the mulberry accessions belonging to section *Alba*, indicating a close relationship among these seven species and three varietas: *M. alba*, *M. atropurpurea*, *M. multicaulis*, *M. mongolica*, *M. bombycis*, *M. mizuho, M. australis*, *M. alba* var. *pendula*, *M. alba* var*. macrophylla*, and *M. mongolica* var. *diabolica* (Table 1). Based on population analyses in mulberry through genome resequencing, Jiao proposed that *M. alba*, *M. multicaulis*, *M. bombycis*, and *M. mizuho* are closely related and should all be classified as *M. alba* [24]. As discussed above, the signal variation mainly on chromosomes 1 and 2 among the accessions in section *Alba* indicated that they differ cytogenetically. In future studies, the genetic relationship between different species in section *Alba* can be further clarified using blocking DNA in GISH, as was done in studies in *Oryza*, *Actinidia*, and *Paphiopedilum* [47–49].

Most of the cultivated mulberries belonged to section *Alba* and were closely related, even though some genetic diversities were detected in this study. The species in sections *Nigra*, *Wittiorum*, and *Cathayana* contained several desirable traits and more distant genetic relationships with species in section *Alba* reflected by GISH signal difference; therefore, sections *Nigra*, *Wittiorum*, and *Cathayana* are important gene pools for mulberry breeding programs. Furthermore, we mostly identified low to medium ploidy levels (mainly diploid) in sections *Alba* (× = 14) and *Notabilis* (× = 7), and medium to high ploidy levels in the wild mulberries in sections *Wittiorum*, *Cathayana*, and *Nigra* (Table 1 and Table S1). Thus, cross breeding between species in section *Alba* and other mulberry sections deserves more attention from breeders to create superior varietas with heterosis and polyploidy advantages.

## Conclusions

We performed cGISH and self-GISH in 40 mulberry accessions from 12 mulberry species and three varietas. We identified six distinct homozygous cGISH signal patterns and one heterozygous cGISH signal pattern. We propose that all mulberry species and varietas investigated here should be classified into five sections. Prominent among them is section *Alba*, which contained seven species and three varietas and could be further divided into two subsections. We also analyzed the genetic relationships between different mulberry species and our results provide guidance for hybridization and polyploid breeding of mulberry.

## Materials and methods

### Plant materials

Twelve mulberry species (*M. notabilis* Schneid, *M. nigra* L., *M. wittiorum* Handelb-Mazz, *M. laevigata* Wall, *M. cathayana* Hemsl, *M. alba* L., *M. multicaulis* Perr, *M. mongolica* C.K. Schneid, *M. atropurpurea* Roxb, *M. bombycis* Koidz, *M. mizuho* Hotta, and *M. australis* Poir) and three varietas (*M. alba* var. *pendula* Dippel, *M. alba* var. *macrophylla* Loud, and *M. mongolica* var. *diabolica* Koidz) were selected for use in this study and represent most of the *Morus* species distributed in China (except *M. serrata* Roxb, *M. nigriformis* Koidz, and *M. alba* var. *venose* Del). The accession name, species name, sample location, ploidy level, and means of propagation of all 40 mulberry accessions used in this study are listed in Supplementary Table 1. All the mulberry accessions were identified by Professor Ningjia He and Doctor Yahui Xuan according to the morphological characteristics, and preserved in Mulberry Germplasm Nursery at Southwest University (N29°49′4.60″, E106°24′35.57″), Chongqing, China.

### Chromosome preparation

Mitotic chromosomes were prepared as described previously with minor modifications [29]. In brief, young leaves or root tips were pretreated with 2 mM 8-hydroxyquinoline at room temperature for 3 h, then fixed in 3:1 ethanol:glacial acetic acid at 4℃ for 4 h. The fixed leaves and root tips were stored in 70% ethanol at -20℃ until use. The leaves and root tips were washed in distilled water for three times. After that, the leaves and root tips were digested in an enzyme solution composed of 2% (w/v) cellulase Onozuka R-10 (YaKult, Japan) and 1% (w/v) pectolyase Y-23 (YaKult, Japan) (pH 5.5) at 37℃ for 3 h and 1 h, respectively. Digested leaves and root tips were rinsed with 70% ethanol and macerated into fine suspensions. The cells were resuspended in glacial acetic acid, and a drop of the suspension was added to a glass slide. Then, the slides were observed using an Olympus IX73 microscope (Olympus, Tokyo, Japan).

### Genomic DNA extraction and probe labeling

Genomic DNA of the 12 mulberry accessions (*M. notabilis*, *M. nigra*, *M. wittiorum* ‘Ailaoshan No. 9’, *M. cathayana* ‘Huai302’, *M. multicaulis* ‘Heyebai’, *M. alba* ‘Baiyuwang’, *M. atropurpurea* ‘Lunjiao109’, *M. alba* var. *pendula* ‘Chuisang’, *M. alba* var. *macrophylla* ‘Dayezaoshengsang’, *M. bombycis* ‘Jianchi’, *M. mongolica* ‘Jimengsang’, and *M. mongolica* var. *diabolica* ‘Taiping No. 5’) used for probe labeling was extracted from young leaves using a DNAquick Plant System kit (TIANGEN BIOTECH, Beijing, China) according to the product manual. 25S rDNA sequences were amplified according to a previous study [29]. Genomic DNA and 25S rDNA probes used for GISH and FISH, respectively, were labeled with ChromaTide Alexa Fluor 488–5-dUTP (Thermo Fisher Scientific [Invitrogen], Massachusetts, USA) or Texas-red-5-dCTP (PerkinElmer, Massachusetts, USA) by a nick-translation method [50]. Briefly, the labelling system included 10 μL of DNA product (containing 2 μg genomic DNA or PCR product of the 25S rDNA sequences), 2 μL nick translation buffer, 2 μL dNTP (-dCTP or -dUTP) mix, 0.5 μL Texas-red-5-dCTP or ChromaTide Alexa Fluor 488–5-dUTP, 0.5 μL DNase I (100 mU/μL), and 5 μL DNA polymerase I (10 U/μL). After incubation at 15℃ for 2 h, the probes were purified in 2.5 volumes of 90% ethanol/10% sodium acetate mix (3 M, pH 5.2), and dissolved with 20 μL 2 × SSC and 1 × TE solution.

### In situ hybridization

Genomic in situ hybridization (GISH) and fluorescence in situ hybridization (FISH) were conducted according to the methods reported by Zhang [50] and Kato [51] with some modifications. In brief, the slides were UV-crosslinked at 1,250 mJ/cm^2^ for 2 min. The probes were diluted with 2 × SSC and 1 × TE to 15 ng/μL, and then added to the slides. The chromosomes and probes were denatured together by heating at 100℃ for 5 min. After overnight hybridization at 42℃, the slides were washed in 2 × SSC at room temperature for 5 min. Chromosomes were counterstained with 1 ng/μL of DAPI and sealed with nail polish. Images were captured with an Olympus IX73 microscope (Olympus Corp., Tokyo, Japan) using the cellSens Standard 1.13 software and a DP80 CCD camera. Images were processed with Adobe Photoshop CS6 and Adobe Illustrator (Adobe, San Jose, CA, USA).

### Supplementary Information

**Additional file 1: Fig. S1.** Karyotype analysis of *M. notabilis* based on the self-genomic in situ hybridization (self-GISH) signal pattern in Fig. 1. **Fig. S2.** Comparative genomic in situ hybridization (cGISH) signal pattern 6 detected in nine mulberry accessions. **Fig. S3.** Comparative genomic in situ hybridization (cGISH) signals detected in nine mulberry accessions. **Fig. S4.** Self-genomic in situ hybridization (self-GISH) and fluorescence in situ hybridization (FISH) signal patterns detected in six mulberry accessions.

**Additional file 2: Supplementary Table 1.** List of the mulberry accessions used in this study.

## Data Availability

All data supporting the findings of this study are available within the paper and within its supplementary materials published online. The plant materials reported in the manuscript are freely available to all the readers on reasonable request.
