# Supplementary material for: Chromosome constitution and genetic relationships of Morus spp. revealed by genomic in situ hybridization
Source: BMC Plant Biol. 2023 Sep 15;23:428. doi: 10.1186/s12870-023-04448-9 (PMC10503058; doi:10.1186/s12870-023-04448-9)
Supplement: Supplementary file 1 — Additional file 1: Fig. S1. Karyotype analysis of M. notabilis based on the self-genomic in situ hybridization (self-GISH) signal pattern in Fig. 1. Fig. S2. Comparative genomic in situ hybridization (cGISH) signal pattern 6 detected in nine mulberry accessions. Fig. S3. Comparative genomic in situ hybridization (cGISH) signals detected in nine mulberry accessions. Fig. S4. Self-genomic in situ hybridization (self-GISH) and fluorescence in situ hybridization (FISH) signal patterns detected in six mulberry accessions. [file 12870_2023_4448_MOESM1_ESM.docx]

**Supplementary Figures**


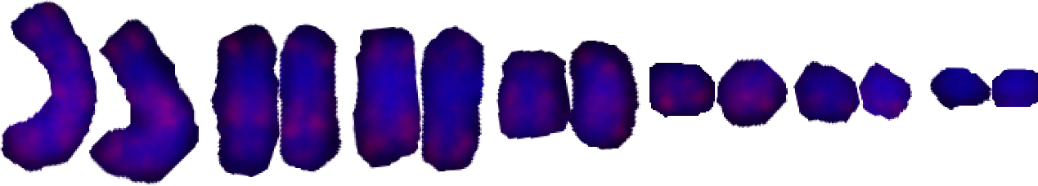


**Fig. S1**. Karyotype analysis of *M. notabilis* based on the self-genomic *in situ* hybridization (self-GISH) signal pattern in Figure 1.


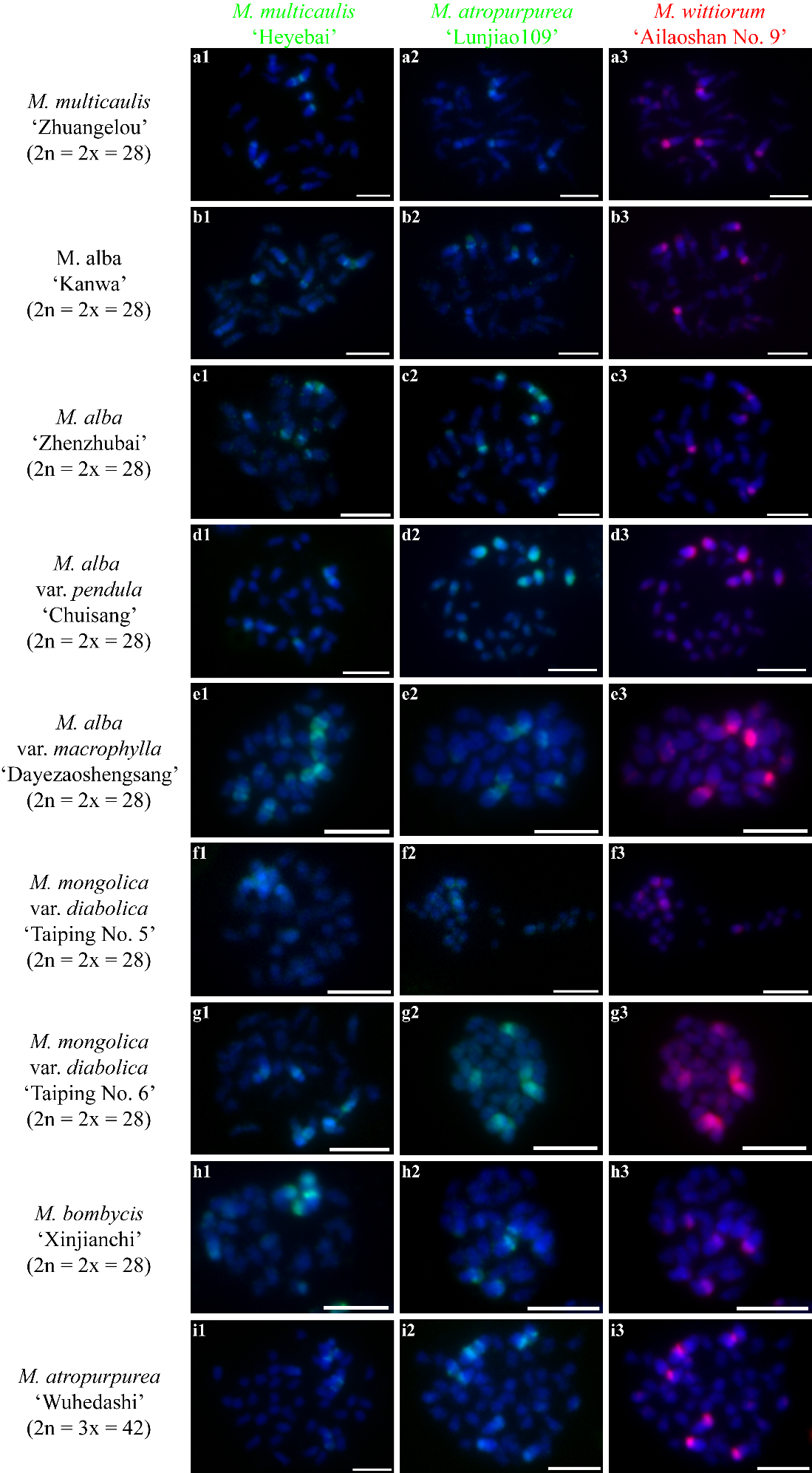


**Fig. S2**. Comparative genomic *in situ* hybridization (cGISH) signal pattern 6 detected in nine mulberry accessions. cGISH signals using genomic DNA of *M. multicaulis* ‘Heyebai’ **(1a–i)**, *M. atropurpurea* ‘Lunjiao109’ **(2a–i)**, and *M. wittiorum* ‘Ailaoshan No. 9’ **(3a–i)** as probes. Scale bars represent 5 μm.


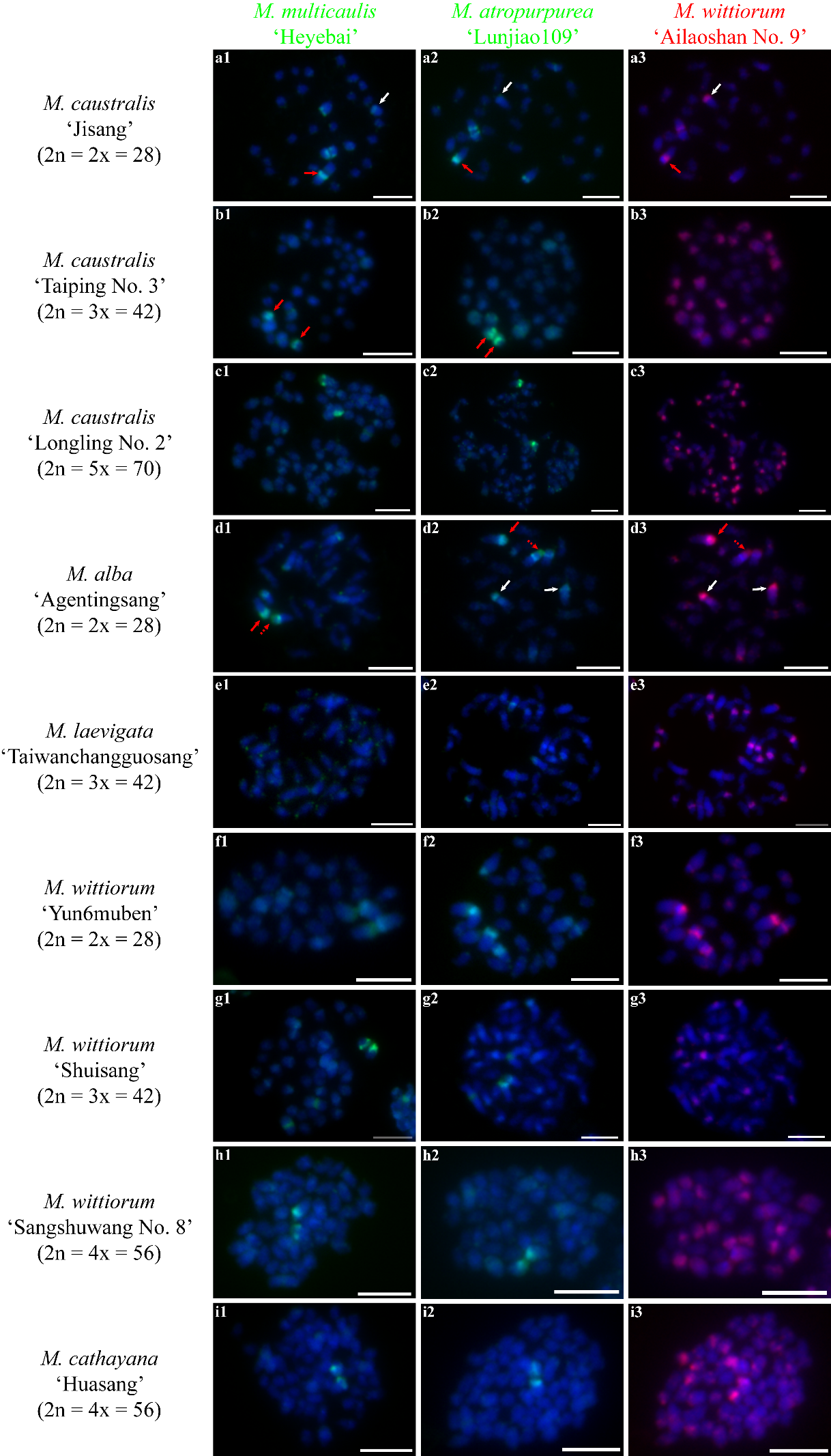


**Fig. S3**. Comparative genomic *in situ* hybridization (cGISH) signals detected in nine mulberry accessions. Heterozygous cGISH signals using genomic DNA of *M. multicaulis* ‘Heyebai’ **(1a–i)**, *M. atropurpurea* ‘Lunjiao109’ **(2a–i)**, and *M. wittiorum* ‘Ailaoshan No. 9’ **(3a–i)** as probes. Red arrows indicate unpaired chromosome 1 (**a1–3, b1–2, d1–3**). White arrows indicate unpaired or paired chromosome 2 with intense signal bands at the short arms (**a1–3, d2–3**). Scale bars represent 5 μm.


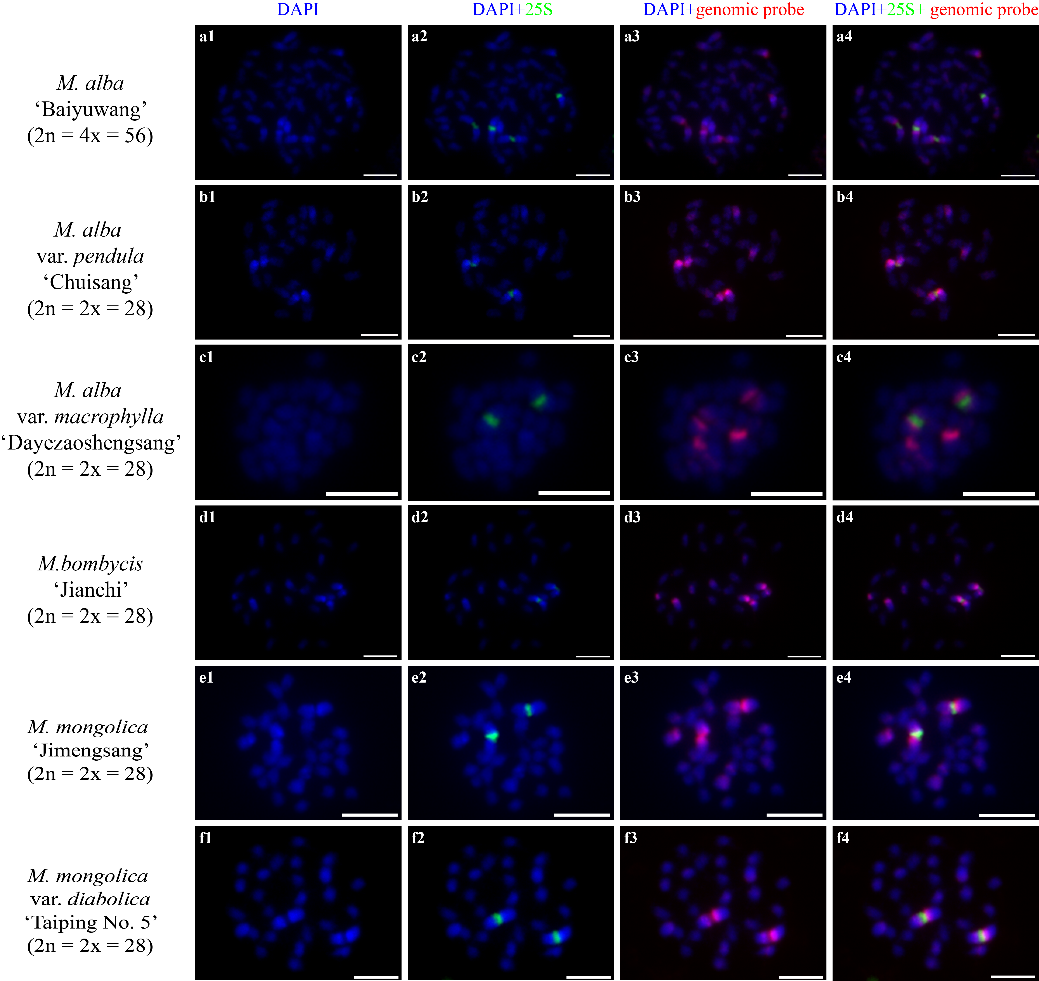


**Fig. S4**. Self-genomic *in situ* hybridization (self-GISH) and fluorescence *in situ* hybridization (FISH) signal patterns detected in six mulberry accessions. (**1a–f)**: chromosomes counterstained with DAPI. **(2a–f)**: FISH signal patterns using the 25S rDNA sequence as probe. **(3a–f)**: self-GISH signal patterns. **(4a–f)**: merged FISH and self-GISH signal patterns. Scale bars represent 5 μm.
